# Supplementary material for: Effects of food type and abundance on begging and sharing in Asian small-clawed otters (Aonyx cinereus)
Source: PeerJ. 2020 Nov 30;8:e10369. doi: 10.7717/peerj.10369 (PMC7713600; doi:10.7717/peerj.10369)
Supplement: Supplementary file 1 [file peerj-08-10369-s002.docx]

GLMM output with outliers removed

Begging and food transfers		*B*	*SE*	*df*	*z*	*p*
**Fixed effects**						
(Intercept)		-2.719	0.158		-17.205	<.001
Begging received		0.147	0.050	1	2.957	**0.003**
**Random effects**		*Variance*				
obs	*(intercept)*	0.152				
ID	*(intercept)*	0.083				
trial	*(intercept)*	0.169				
Observation	169					
ID	20					
