# Supplementary material for: Effects of food type and abundance on begging and sharing in Asian small-clawed otters (Aonyx cinereus)
Source: PeerJ. 2020 Nov 30;8:e10369. doi: 10.7717/peerj.10369 (PMC7713600; doi:10.7717/peerj.10369)
Supplement: Supplementary file 2 [file peerj-08-10369-s003.docx]

GLMM output without outliers

Begging		*B*	*SE*	*df*	*z*	*p*
Fixed effects						
(Intercept)		-2.790	0.327		-8.543	<.001
Food type	crab	0	0			
	mussel	-0.522	0.289	2	-1.803	0.071
	trout	-0.221	0.291	2	-0.759	0.448
Food density	low	0	0			
	medium	-0.022	0.282	2	-0.079	0.937
	high	-0.551	0.294	2	-1.887	0.061
Age category	adult	0	0			
	juvenile	0.233	0.174	1	1.337	0.181
Sex	female	0				
	male	0.002	0.175	1	0.013	0.990
Zoo	Newquay	0	0			
	Tamar	0.271	0.240	1	1.132	0.258
Random effects		*Variance*				
obs	*(Intercept)*	0.283				
ID	*(Intercept)*	0.140				
trial	*(Intercept)*	0.109				
Observation	168					
ID	20					
