# Supplementary material for: Effects of food type and abundance on begging and sharing in Asian small-clawed otters (Aonyx cinereus)
Source: PeerJ. 2020 Nov 30;8:e10369. doi: 10.7717/peerj.10369 (PMC7713600; doi:10.7717/peerj.10369)
Supplement: Supplementary file 3 [file peerj-08-10369-s004.docx]

GLMM output without outliers

Sharing		*B*	*SE*	*df*	*z*	*p*
Fixed effects						
(Intercept)		-2.466	0.348		-7.081	<.001
Food type	crab	0	0			
	mussel	-0.260	0.271	2	-0.960	0.337
	trout	0.068	0.281	2	0.242	0.809
Food density	low	0	0			
	medium	0.392	0.277	2	1.413	0.158
	high	0.189	0.281	2	0.672	0.502
Age category	adult	0	0			
	juvenile	-0.792	0.208	1	-3.803	**<0.001**
Sex	female	0				
	male	-0.200	0.212	1	-0.945	0.345
Zoo	Newquay	0	0			
	Tamar	-0.111	0.274	1	-0.404	0.686
Random effects		*Variance*				
obs	*(Intercept)*	0.107				
ID	*(Intercept)*	0.094				
trial	*(Intercept)*	0.115				
Observation	179					
ID	20					
